# The Influence of Recombinant Production on the Immunologic Behavior of Birch Pollen Isoallergens

**DOI:** 10.1371/journal.pone.0008457

**Published:** 2009-12-24

**Authors:** Michael Wallner, Martin Himly, Angela Neubauer, Anja Erler, Michael Hauser, Claudia Asam, Sonja Mutschlechner, Christof Ebner, Peter Briza, Fatima Ferreira

**Affiliations:** 1 Christian Doppler Laboratory for Allergy Diagnosis and Therapy, University of Salzburg, Salzburg, Austria; 2 Biomay AG, Vienna Competence Center, Vienna, Austria; 3 Christian Doppler Laboratory for Immunemodulation, Medical University of Vienna, Vienna, Austria; 4 Allergieambulatorium, Vienna, Austria; 5 Department of Molecular Biology, University of Salzburg, Salzburg, Austria; Monash University, Australia

## Abstract

**Background:**

Allergic reactions towards the birch major pollen allergen Bet v 1 are among the most common causes of spring pollinosis in the temperate climate zone of the Northern hemisphere. Natural Bet v 1 is composed of a complex mixture of different isoforms. Detailed analysis of recombinant Bet v 1 isoforms revealed striking differences in immunologic as well as allergenic properties of the molecules, leading to a classification of Bet v 1 isoforms into high, medium, and low IgE binding proteins. Especially low IgE binding Bet v 1 isoforms have been described as ideal candidates for desensitizing allergic patients with allergen specific immunotherapy (SIT). Since diagnosis and therapy of allergic diseases are highly dependent on recombinant proteins, continuous improvement of protein production is an absolute necessity.

**Methodology:**

Therefore, two different methods for recombinant production of a low IgE binding Bet v 1 isoform were applied; one based on published protocols, the other by implementing latest innovations in protein production. Both batches of Bet v 1.0401 were extensively characterized by an array of physicochemical as well as immunological methods to compare protein primary structure, purity, quantity, folding, aggregation state, thermal stability, and antibody binding capacity.

**Conclusion:**

The experiments demonstrated that IgE antibody binding properties of recombinant isoallergens can be significantly influenced by the production method directly affecting possible clinical applications of the molecules.

## Introduction

IgE-mediated hypersensitivity reactions, the most common cause of allergy, affect more than 25% of the population in industrialized countries. Typically, the allergic response is directed against environmental proteins, so called allergens that originate from various sources including pollen, spores of moulds, food, mites, cockroaches, and animal dander. The allergic disease is typically divided into a sensitization phase, where patients do not experience allergic symptoms but become primed to react with certain allergenic molecules, and the immediate phase of the allergic reaction. During sensitization allergen-specific B cells are activated to produce and secrete IgE antibodies, which attach via the high affinity receptor FcεRI to the surface of mast cells and basophils. Upon repeated allergen contact, cross-linking of the IgE-FcεRI on mast cells and basophils triggers degranulation and release of vasoactive amines (*i.e.* histamine), lipid mediators (*i.e.* prostaglandins, leukotrienes), chemokines, and cytokines. These events characteristic for the immediate phase of the allergic reaction cause the typical allergic symptoms, *i.e.* allergic rhinitis, conjunctivitis, hay fever or asthma, eczema, allergen elicited gastrointestinal diseases, and anaphylaxis [1], [2]. Specific immunotherapy (SIT) constitutes the only curative approach to treat allergic diseases. During SIT increasing doses of allergen are administered to a patient, ideally followed by modulation of the allergic immune response at the levels of antigen presenting cells, T cells and B cells. Molecules suitable for SIT should address all these arms of the immune system, and still not elicit adverse side effects. To increase the safety profile of SIT the use of well-standardized low IgE-binding recombinant allergens seems most preferable [3], [4]. Allergic reactions towards the birch major pollen allergen Bet v 1 are among the most common causes of spring pollinosis in the temperate climate zone of the Northern hemisphere. Natural Bet v 1 represents a complex mixture of different isoforms and to date more than 30 have been identified showing differences concerning allergenicity as well as immunogenicity [5], [6], [7]. Formerly, the isoforms have been termed alphabetically from Bet v 1a to n, which has been replaced by a numbering system appointing the isoforms from Bet v 1.0101 to Bet v 1.3001. Furthermore, a detailed immunologic characterization led to a classification of the Bet v 1 family in high, medium, and low IgE binding molecules [8], [9]. Based on this data, naturally occurring low IgE binding Bet v 1 isoforms appeared most suitable for safe application in SIT. In this context, the low IgE binding birch pollen isoform Bet v 1.0401, formerly Bet v 1d, was most detailed investigated [9]. Notably, all studies of Bet v 1 isoforms have been performed with recombinant proteins, however, external influences such as production, storage condition, or buffer systems can manipulate the allergenic but also immunologic properties of the molecules. For instance, thermal processing of Bet v 1 results in unfolding of the molecule. This loss of the native structure is accompanied by a loss of its IgE binding capacity [10]. Since IgE binding to Bet v 1 is highly fold-dependent, production procedures can have direct impact on the allergenic properties of the recombinant allergen preparation. Recently, Kahlert *et* al. rendered the *per se* high IgE binding Bet v 1.0101 into a hypoallergenic folding variant by treating the protein with basic buffer during recombinant production [11].

The aim of the present work was to investigate how latest innovations in protein production could influence the allergenic properties of recombinant Bet v 1.0401 by comparing two different production protocols. In view of the conformation-dependent IgE binding properties of Bet v 1, the study focused on investigating expression and purification parameters directly impacting protein folding. Thus, one batch of Bet v 1.0401 was produced from a cDNA clone of birch pollen RNA and expressed at 37°C without further optimization according to previously published methods with minor modifications [12], [13], [14]. The overall yields of recombinant Bet v 1.0401 appeared adequate; however, the protein had to be purified from bacterial inclusion bodies and refolded during purification. Alternatively, an expression and purification protocol based on an *E. coli* codon optimized *Bet v 1.0401* gene was established. Low temperature expression led to increased amounts of soluble protein allowing protein purification without a denaturation/renaturation step. Both production batches were analyzed by an array of physicochemical methods for primary structure, purity, quantity, folding, aggregation state, and thermal stability [15] as well as immunologically investigating antibody binding on solid surface and in solution.

## Material and Methods

### Patients and Sera

*Fagales* pollen-allergic patients were selected on the basis of typical case history, positive *in vivo* skin prick test, and *in vitro* IgE detection (CAP system, Phadia AB, Uppsala, Sweden). Patients with IgE values greater than class 3 were selected. The study was approved by the Local Ethic Committee of the University of Salzburg, and informed written consent was obtained from all subjects included in the study.

### Recombinant Reference Allergens

Recombinant Bet v 1.0101 (X15877), also known as Bet v 1a, which shows an amino acid sequence identity of 95% with Bet v 1.0401, formerly termed Bet v 1d, was used as reference allergen. The protein was purchased from Biomay AG (Vienna, Austria).

### Bacterial Strains and Plasmids

For protein expression of Bet v 1.0401 (X77266) two constructs were designed. Both constructs encoded for the same protein. For the first construct the DNA sequence of *Bet v 1.0401* obtained by cDNA cloning of the birch pollen allergen from its natural source was used. The second construct is based on an *E. coli* codon optimized gene of *Bet v 1.0401*. Both genes were inserted into the T7-based expression vector pET28b (Novagen, Merck Chemicals Limited, Nottingham, UK). The *E. coli* strain BL21 (DE3) (Stratagene, La Jolla, CA, USA) was used for production of recombinant proteins.

### Expression and Purification of Recombinant Bet v 1.0401 - Production Batch A

Bet v 1.0401 batch A was produced according to previously published methods with minor modifications [12], [13], [14]. Therefore, the cDNA of *Bet v 1.0401* obtained from a birch pollen library was inserted into the vector pET28b was used for production of recombinant protein. Expression plasmids were freshly transformed and *E. coli* BL21 (DE3) cells were grown in LB medium supplemented with 25 mg/L kanamycin at 37°C to an OD_600_ of 0.8. After addition of 0.5 mM IPTG, expression of Bet v 1.0401 was performed for 6 h at 37°C. Cells were harvested by low speed centrifugation; inclusion bodies were prepared and resuspended in 10 mM imidazole pH 7.4, 100 mM NaCl, supplemented with 10 mM dithioerythritol (DTE) to reduce intermolecular interactions of Bet v 1.0401 via its single cysteine. Proteins were solubililzed by addition of 10 M NaOH under constant stirring until a pH of 13 was reached. After 5 min incubation citric acid was added to lower the pH to 8.6. The solution was centrifuged at 18.000 g for 10 min followed by addition of 20 mM DTE and incubation 1 h at 37°C. Afterwards solid NaCl and NaH_2_PO_4_ were added under constant stirring on ice to final concentrations of 1 M and 0.2 M, respectively. Bet v 1.0401 was purified by hydrophobic interaction chromatography using a 100 ml Phenyl sepharose column (GE Healthcare, Little Chalfont, UK). The protein was eluted with 25 mM Tris/HCl pH 9.3, 8% (v/v) 2-propanol and resulting fractions containing Bet v 1.0401 were pooled and dialyzed against 10 mM sodium phosphate pH 7.2, 2 mM β–mercaptoethanol. Final purification was performed by HPLC using a Hypersil C-8 column (Thermo Fisher Scientific Inc., Waltham, MA, USA) with a flow rate of 5 ml/min. Bet v 1.0401 was eluted using a 60 ml gradient 0–80% buffer B from 0.1% trifluoroacetic acid to 0.1% trifluoroacetic acid, 90% (v/v) 2-propanol (Table 1). Purified recombinant protein was dialyzed against 10 mM sodium phosphate buffer pH 7.4, freeze dried, and stored at −20°C.

**Table 1:** Recombinant production of Bet v 1.0401.

	Production batch A	Production batch B
Gene	*Bet v 1.0401*, plant codon usage	*Bet v 1.0401*, *E. coli* codon usage
Expression plasmid, cloning sites	pET28b, 5′ Nco I, 3′ Eco RI	pET28b, 5′ Nco I, 3′ Eco RI
Bacterial cells	BL21 (DE3)	BL21 Star™ (DE3)
Expression conditions	37°C, 6 h	16°C, 18 h
Extraction	inclusion bodies with NaOH, pH 13	soluble proteins with 0.5 M Urea
Chromatography	HIC, reversed phase HPLC	HIC, IEX

### Expression and Purification of Recombinant Bet v 1.0401 - Production Batch B

Codon-optimized *Bet v 1.0401* inserted into the vector pET28b was used for production of recombinant protein. Expression plasmids were freshly transformed and *E. coli* Star™ BL21 (DE3) (Invitrogen, Paisley, UK) cells grown at 37°C in 4L LB supp. medium (1% (w/v) peptone, 0.5% (w/v) yeast extract, 0.5% (w/v) NaCl, 2 mM MgSO_4_, 1% (v/v) glycerol, 0.2% (w/v) ammonium sulfate, 10 mM sodium phosphate pH 7.4) supplemented with 25 mg/L kanamycin at 37°C to an OD_600_ of 0.8. After addition of 0.5 mM IPTG expression of Bet v 1.0401 was performed for 18 h at 16°C. Cells were harvested by low speed centrifugation and resuspended in 25 mM sodium phosphate buffer pH 7.4, 0,1% (v/v) Triton X-100, 0.5 M urea. Additionally, solid NaCl and NaH_2_PO_4_ were added under constant stirring on ice to final concentrations of 1 M and 0.5 M, respectively. After centrifugation at 15.000 g, the solution was filtered through a 0.45 µm filter and soluble Bet v 1.0401 was purified by hydrophobic interaction chromatography using a 100 ml Phenylsepharose column (GE Healthcare). Urea was removed by applying a gradient of 200 ml using 0.2 M NaH_2_PO_4_, 1 M NaCl, pH 4.2. The protein was eluted with 25 mM Tris/HCl pH 9.3, 8% (v/v) 2-propanol and resulting fractions containing Bet v 1.0401 were pooled and dialyzed against 20 mM imidazole pH 7.4, 4% (v/v) 2-propanol. Final purification was performed by anion exchange chromatography using a 100 ml DEAE sepharose column (GE Healthcare). Bet v 1.0401 was eluted with 20 mM imidazole pH 7.4, 4% (v/v) 2-propanol, 250 mM NaCl using a 1200 ml gradient. All chromatographic steps were performed with an ÄKTA Prime (GE Healthcare) using a flow rate of 2.2 ml/min (Table 1). Purified recombinant protein was dialyzed against 10 mM sodium phosphate buffer, pH 7.4, freeze dried, and stored at −20°C.

### SDS-PAGE

*E. coli* lysates as well as purified proteins were analyzed by sodium dodecyl sulfate polyacrylamide gel electrophoresis (SDS-PAGE) according to the method described by Laemmli [16], using either 15% gels or alternatively 12–24% gradient gels. Proteins were visualized by staining with Coomassie Brilliant Blue R-250 (Biorad, Hercules, CA, USA).

### Physicochemical Characterization of Recombinant Proteins

Protein identity was determined by mass spectrometry using a Quadrupole time-of-flight mass spectrometer with electrospray ionization (ESI-QTOF-MS) (Waters Corp., Milford, MA, USA). Therefore, proteins were separated by 2D gel electrophoresis and Coomassie-stained spots were excised from 2D gels [17]. Spots were analyzed by densitometry using Photoshop CS3 (Adobe Systems Incorporated, San Jose, CA, USA). Intact proteins were extracted and directly infused into a Global Ultima Q-TOF instrument (Waters Corp., Milford, MA, USA) with electrospray ionization [17]. Protein secondary structure content was determined by circular dichroism (CD) spectroscopy. CD spectra were recorded with a JASCO J-815 spectropolarimeter fitted with a PTC-423S Peltier type single position cell holder in appropriate buffers (Jasco, Tokyo, Japan). Homogeneity and aggregation behaviour in solution of Bet v 1.0401 were performed by online high performance-size exclusion chromatography (HPSEC)–light scattering and dynamic light scattering (DLS), respectively, as described elsewhere [15].

### Fluorescence Spectroscopy

8-anilino-1-naphtalenesulfonic acid (ANS) binding experiments with recombinant allergens were performed using 10 µM of the respective allergen and increasing concentrations of ANS, preparing aliquots for each data point. Fluorescence measurements were performed in 5 mM sodium phosphate buffer pH 7.4 at 450 nm using an excitation wavelength of 370 nm in a Tecan Infinite 200 microplate reader (Tecan Group Ltd., Männedorf, Switzerland) [18]. Nonlinear regressions of ligand binding data as well as dissociation constants (Kd) were calculated using Sigmaplot 11.0 (Systat Software Inc., San Jose, CA, USA).

### ELISA Experiments

Maxisorp plates (NUNC, Wiesbaden, Germany) were coated with titrations 2 µg/ml protein in PBS in a total volume of 50 µl overnight at 4°C. After washing and blocking, patients' sera diluted 1 to 5 in 50 µl were added overnight at 4°C. Bound IgE was detected with alkaline phosphatase-conjugated monoclonal anti-human IgE antibodies (BD Biosciences, Franklin Lakes, NJ, USA) in a colorimetric assay. All measurements were performed as triplicates. Results are presented as mean OD values after baseline correction.

### Mediator Release from Rat Basophilic Leukemia Cells (RBL Cells)

The allergenic potential of recombinant pollen allergens was measured by degranulation assays using rat basophil leukemia cells RBL-2H3 transfected with the human high-affinity IgE receptor (FcεRI). Briefly, RBL-2H3 cells, were passively sensitized with human serum IgE from birch pollen-allergic patients. After washing, degranulation was triggered by addition of serial dilutions of the respective allergens. β-hexosaminidase release into the supernatant was measured by enzymatic cleavage of the fluorogenic substrate 4-methylumbelliferyl-N-acetyl-β-D-glucosaminide and expressed as % of total enzyme content of Triton X-100-treated cells [19].

### Statistical Analysis

Statistical evaluation of ELISA experiments with human serum samples (*n* = 13) and rat basophil mediator release assays using sera of allergic individuals (*n* = 8) were calculated with paired samples *t*-test. A value of *P*<0.05 was considered statistically significant.

## Results

### Production Batch A - Purification of Bet v 1.0401 under Denaturing Conditions Followed by Refolding

**Figure 1:**
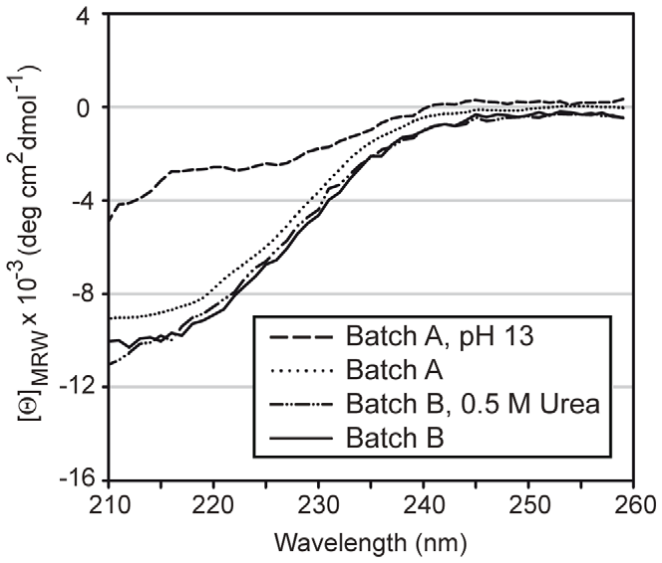
Circular dichroism analysis of Bet v 1.0401. CD spectra of Bet v 1.0401 batch A were recorded in 10 mM sodium phosphate after addition of sodium hydroxide pH 13 (dashed line) and after refolding in 10 mM sodium phosphate pH 7.4, Spectra of batch B were recorded in 10 mM sodium phosphate pH 7.4, 0.5 M Urea (dashed dot dotted line) and after removal of the Urea in 10 mM were recorded in 10 mM Data are presented as mean residue molar ellipticity [Θ]_MRW_ at a given wavelength and baseline corrected. For all measurements quartz cuvettes with a path length of 0.1 cm were used. Far UV CD spectra were recorded at 20°C, with a band width of 1 nm, a response time of 1 sec and a data pitch of 1 nm. The measurement range was between 190–260 nm. Each spectrum represents an average of five consecutive scans.

**Figure 2:**
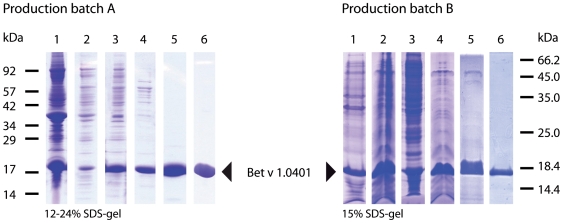
Purification of Bet v 1.0401. SDS-PAGE analysis of Bet v 1.0401 purification. Bet v 1.0401 batch A was purified from E. coli lysates using a denaturing step during production, batch B was purified from soluble bacterial lysate. Production batch A, lane 1, total bacterial lysate; lane 2, soluble bacterial lysate; lane 3, fraction after basic denaturation; lane 4, salt precipitated fraction; lane 5 hydrophobic interaction chromatography; lane 6, reversed phase chromatography. Production batch B, lane 1, total bacterial lysate; lane 2, soluble bacterial lysate; lane 3, salt precipitated pellet; lane 4, salt precipitated fraction; lane 5, hydrophobic interaction chromatography; lane 6, anion exchange chromatography.

Recombinant Bet v 1.0401 was expressed from the plasmid pET28b as non-fusion protein in *E. coli* BL21 (DE3) at 37°C. After disruption of the cells, Bet v 1.0401 was found in inclusion bodies, which were dissolved by addition of 10 M sodium hydroxide to increase the pH of the solution to 13. This resulted in denaturation of Bet v 1.0401 (Figure 1), however, the protein refolded after lowering the pH to 8.5 using citric acid. Thereafter, Bet v 1.0401 was purified by hydrophobic interaction chromatography followed by a polishing step using reversed phase chromatography on a HPLC (Figure 2). 1 liter *E. coli* culture yielded 38 mg pure Bet v 1.0401.

### Production Batch B - Purification of Bet v 1.0401 without Denaturation/Renaturation Results in Improved Solubility and Enhanced Protein Yields

To increase the yield of recombinant Bet v 1.0401 in *E. coli* especially at low expression temperature, a codon-optimized gene was designed [20], integrated in a pET28b plasmid, and transformed into BL21 Star™ (DE3) cells. This *E. coli* strain has been designed to further improve protein yields of T7-based expression systems. Cells were grown at 37°C in modified LB medium to an OD_600_ of 0.8 in shaking flasks and after induction protein expression was performed at 16°C for 18 h [21]. In contrast to batch A, where Bet v 1.0401 was deposited in classical inclusion bodies by *E. coli* cells, low-temperature production of Bet v 1.0401 resulted in the formation of non-classical inclusion bodies. Unlike classical inclusion bodies, where inactive recombinant proteins form large aggregates, non-classical inclusion bodies constitute of large amounts of most likely correctly folded over-expressed protein [22]. Bet v 1.0401 batch B was extracted from such non-classical inclusion bodies under mild conditions by addition of 0.5 M urea, which did not introduce any change in the molecule's fold (Figure 1). Further purification was performed using hydrophobic interaction and anion exchange chromatography (Figure 2). Applying the optimized production method, 1 liter *E. coli* culture yielded 80 mg pure Bet v 1.0401.

### Different Production Methods Directly Influence the Primary Structure of Bet v 1.0401

**Figure 3:**
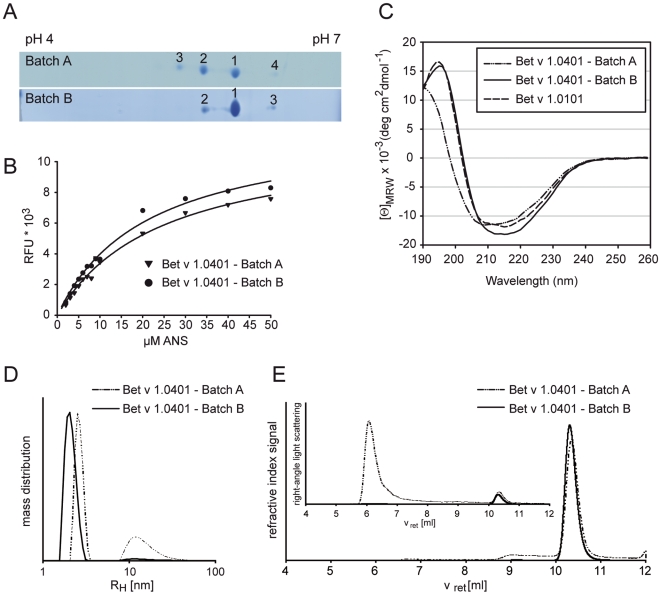
Physicochemical characterization of Bet v 1.0401. Bet v 1.0401 batches were separated by 2D gel electophoresis. Gels were coomassie-stained, numbered spots were analyzed by MS and assigned to respective charge variants (A). ANS titration curves were recorded in 10 mM sodium phosphate (pH 7.4) using excitation and emission wavelengths of 370 and 450 nm, respectively. Solid lines represent nonlinear regressions of the experimental data (B). Circular dichroism spectra are presented as mean residue molar ellipticity [Θ]_MRW_ at a given wavelength and baseline corrected. Bet v 1.0101 was used as reference for Bet v 1-like fold (C). Aggregation behaviour of Bet v 1.0401 batches was investigated by dynamic light scattering (DLS) in aqueous solution at a concentration of 1 mg/ml (D) and by online HPSEC-light scattering analysis operated at 0.5 ml/min in 0.1 M sodium phosphate pH 6.5, 150 mM sodium chloride (E). Analysis of Bet v 1.0401 batch A with DLS showed higher aggregation tendency (66% RH of 2.6±0.3 nm and 34% RH of 16±8 nm) compared to batch B (98% RH of 2.1±0.3 nm and 2% RH of 16±10 nm) (D). In online HPSEC-light scattering of batch A, molecular weight values of 70–80 kDa and 17 kDa were determined from refractive index and right-angle light scattering signals from peaks of oligomeric and monomeric Bet v 1.0401 eluting at retention times (vret) of 9.1 and 10.3 ml, respectively. For batch B one peak at vret of 10.3 ml with a molecular weight of 17 kDa was detected.

As posttranslational modifications often influence the pI of proteins, thus, resulting in multiple spots of differently charged variants of the same protein, both Bet v 1.0401 batches were evaluated by 2D gel electrophoresis. Batch A of Bet v 1.0401 resulted in 4 spots: (i) spot 1 with a relative abundance of 47.2% of total Bet v 1.0401 showed an average mass of 17417.2 Da, whereas (ii) spot 2 (37.8%) and (iii) spot 3 (12.5%) showed a distinct mass shift of +1 (17418.2 Da) and +2 Da (17419.2 Da), respectively. These mass shifts are the result of deamidation of Asn frequently observed for members of the Bet v 1 family [15], [23]. Spot 4 accounted for 2.5% of Bet v 1.0401 batch A. According to the pI of this spot a negative charge of Bet v 1.0401 was either masked or removed, which could be a result of either a C-terminal modification or a conformer of Bet v 1.0401. However, peptide sequencing of the corresponding spot could not detect any modification (sequence coverage of 85,5%). In case of batch B, only 3 spots were visible on the 2D gel: (i) spot 1 with the correct mass of 17417.2 Da containing 72.5% and (ii) spot 2 with a mass shift of +1 Da (17418.2 Da) containing 22.6% of Bet v 1.0401. Spot 3, which corresponded to spot 4 of batch A, accounted for 4.9% of Bet v 1.0401 batch B (Figure 3).

### Bet v 1.0401 Produced under Denaturing Conditions Forms High Molecular Weight Aggregates

An important parameter for quality assessment of protein solutions is represented by the aggregation behavior, which was investigated for both Bet v 1.0401 production batches using online HPSEC-light scattering and DLS. When subjected to chromatography several fractions could be resolved for Bet v 1.0401 production batch A reflecting different aggregation states of the protein (Figure 3). Interestingly, production under denaturing conditions resulted in formation of 2% multimers with a MW of more than 2,000 kDa, 12% small oligomers of 70–80 kDa (primarily tetramers), and >80% monomers. The aggregation behavior of production batch A was even more pronounced when investigated by DLS, a batch technique that does not disrupt potentially reversible aggregates due to shear forces or diluting effects characteristic for high pressure chromatographic techniques. Using DLS, one third of high MW aggregates (>2,000 kDa) was observed for this batch. The other peak at a hydrodynamic radius of 2.6 nm resulted from an unresolved mixture of monomers and small oligomers, which was also detected by HPSEC. In contrast, production conditions avoiding a denaturation/renaturation step prevented an elevated aggregation tendency. More than 99% of Bet v 1.0401 production batch B appeared monomeric in online HPSEC-light scattering and less than 3% of high MW aggregates were determined by DLS experiments of Bet v 1.0401 production batch B.

### Secondary Structure Elements and Folding Properties of Bet v 1.0401 Batches Revealed Remarkable Differences

Secondary structure elements of recombinant proteins were analyzed by CD spectroscopy. Since natural Bet v 1.0401 can only be obtained in minute amounts from birch pollen and therefore has never been purified no reference CD is available for the allergen. Thus, the CD spectra of both Bet v 1.0401 production batches were compared to the spectrum obtained from Bet v 1.0101, an isoform which was demonstrated to produce identical CD spectra as natural Bet v 1 and therefore, providing an ideal reference candidate [11]. The CD spectrum of Bet v 1.0401 batch B was comparable to the spectrum obtained from Bet v 1.0101. However, the spectrum obtained from Bet v 1.0401 batch A, which has been denatured for protein purification, showed a distinct shift of the CD curve intersection at 0 [Θ]_MRW_ units from 202 to 198 nm (Figure 3). This might be an indication for a slightly alternative fold of batch A, or for a mixture of folded an unfolded proteins coexisting in this production batch. Next, the melting points (Tm) were calculated for both batches of Bet v 1.0401. Therefore, the protein was progressively heated with a temperature slope of 1°C/min and the unfolding of Bet v 1.0401 was monitored at a wavelength of 222 nm. Bet v 1.0401 produced without a denaturation/renaturation step showed a Tm of 61.70±0.31°C, whereas denaturation and renaturation of Bet v 1.0401 during recombinant production resulted in a 3.15°C lower Tm of 58.55±0.63°C.

### Recombinant Production Can Influence Ligand Binding Properties of Bet v 1.0401

Bet v 1 has a Y-shaped, solvent accessible hydrophobic cavity traversing the core of the molecule, capable of binding a wide range of ligands. 8-anilino-1-naphtalenesulfonic acid (ANS), a substrate, which is essentially non-fluorescent, however, displays fluorescence when bound to hydrophobic patches of proteins, has been reported to bind this cavity of Bet v 1 [24], [25]. Experiments revealed that ANS binds to both production batches of Bet v 1.0401 with a binding stoichiometry of 2 to 1. However, the dissociation constant (Kd) was increased for ligand binding of batch A (Kd = 24.46 µM) when compared to batch B (Kd = 21.23 µM) (Figure 3). This observation could be a result of the tendency of batch A to form high molecular weight aggregates, which could obstruct the access to the cavity and hamper ligand binding.

### Different Production Methods Directly Affect IgE Binding Properties of Bet v 1.0401

**Figure 4:**
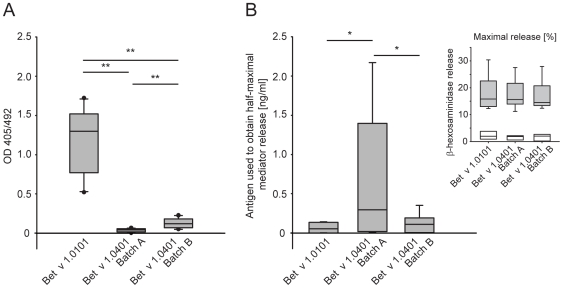
Allergenic activity of Bet v 1.0401. IgE binding properties of Bet v 1.0401 batches were tested by ELISA. Results are presented as mean OD values of triplicate measurements after background subtraction. Sera of 13 birch pollen-allergic individuals were tested. Data are presented as median OD405/492 units, 10^th^, 25^th^, 75^th^, and 90^th^ percentiles as vertical boxes with error bars. *P*-values were calculated by paired-samples *t*-test (**P<.01) (A). Allergenic activity of Bet v 1.0401 batches was assessed in huFcεRI-transfected RBL-2H3 cells sensitized with serum IgE from birch pollen-allergic patients (n = 8). Protein concentrations (ng/ml) to obtain 50% of maximum degranulation were determined for the respective antigens. *P*-values were calculated by paired-samples *t*-test (*P<.05). In addition, protein concentrations (ng/ml) to obtain maximal mediator release was determined for the respective antigens, as indicated in the upper right corner of the figure grey bars. As negative control RBL cells were loaded with normal human serum (n = 3) and activated with the respective antigens as indicated by white bars. All data are presented as median, 10^th^, 25^th^, 75^th^, and 90^th^ percentiles as vertical boxes with error bars (B).

To investigate IgE antibody binding of Bet v 1.0401 ELISA experiments with sera from 13 birch pollen-allergic patients were performed. Compared to the high IgE binding reference allergen Bet v 1.0101, both production batches of Bet v 1.0401 showed significantly reduced binding of serum IgE (*P*<0.01) (Figure 4). Interestingly, Bet v 1.0401 batch A did not show any IgE binding activity, whereas Bet v 1.0401 batch B bound significantly more serum IgE (*P*<0.01). The altered structure of batch A could facilitate further distortion of the molecule upon binding to the surface of the ELISA plate, which in consequence, could hinder patients' serum IgE binding in the assay.

### Recombinant Bet v 1.0401 Produced under Denaturing Conditions Shows Lower Allergenic Activity

Rat basophils loaded with sera from birch pollen-allergic patients (*n* = 8) were used to determine the allergenic activity of the two Bet v 1.0401 batches. Allergen amounts necessary to trigger half maximal release of inflammatory mediator were determined. The half maximal mediator release per patient was defined by the dose-dependent mediator release curve of rBet v 1.0101, which was used as high IgE binding reference allergen. Bet v 1.0401 batch B showed only a 2 fold decreased allergenic activity when compared to Bet v 1.0101. However, Bet v 1.0401 batch A displayed an 11-fold (*P*<0.05) reduced IgE binding activity when compared to Bet v 1.0101 and a 6-fold (*P*<0.05) reduced activity when compared to Bet v 1.0401 batch B (Figure 4). Though, the three allergens could elicit comparable maximal mediator release of the sensitized basophils.

## Discussion

As alternative production procedures can have dramatic effects on protein folding, during the present work two different recombinant preparations of the birch pollen allergen isoform Bet v 1.0401 were analyzed. Batch A based on a denaturing step during purification, applying a method previously published for the purification of Bet v 1 isoforms with minor modifications [12], [13], [14], and batch B prepared from soluble bacterial lysate following an optimized production protocol. An array of physicochemical as well as immunological investigations to analyze identity, quantity, homogeneity, folding and aggregation behavior, as well as biological activity was applied to both Bet v 1.0401 batches.

Analysis of the primary structure of Bet v 1.0401 using a combination of isoelectric focusing and mass spectrometry revealed batch A to be composed of a mixture of 4 differently charged Bet v 1.0401 species, amongst others being a result of deamidation of Asn. Interestingly, the vast majority of batch B consisted of a single protein species showing no posttranslational modifications. Bet v 1.0401 batch A showed a clear tendency to form high molecular weight aggregates ranging from smaller oligomers (12%) to large multimeric complexes (2%), whereas more than 99% of batch B appeared monomeric when analyzed by online HPSEC-light scattering. CD spectroscopy revealed changes in secondary structure of Bet v 1.0401 batch A, which also manifested in a lower melting point of the protein. These changes in the secondary structure might provide an explanation for increased aggregation of the allergen. In contrast, batch B showed a CD spectrum typical for Bet v 1. Crystallization of several Bet v 1 isofoms revealed that the core of the protein is traversed by Y-shaped, solvent accessible, hydrophobic cavity capable of binding a wide range of ligands [24], [25], [26]. Therefore, the ability of ligand binding was investigated using the fluorescent substrate ANS. The dissociation constant (Kd) for ANS was shown to be higher in Bet v 1.0401 batch A than in batch B, which might be again an indication of fold changes of the protein. Additionally, patients' serum IgE binding was assessed in ELISA experiments including Bet v 1 isoform 0101, which was demonstrated to be suitable as reference material for analysis of IgE binding to Bet v 1 [27], as positive control. Both batches of Bet v 1.0401 showed a significantly reduced IgE binding capacity when compared to Bet v 1.0101 (32-fold versus 9-fold reduction for batch A and B, respectively). This is explainable, since Bet v 1.0401 represents a low IgE binding Bet v 1 isoform, whereas Bet v 1.0101 is a strong IgE binding molecule [7]. Still, Bet v 1.0401 batch B could bind patients serum IgE to some extent, however, IgE binding to batch A was not detectable. This could be confirmed by basophil mediator release assays demonstrating double the amount of Bet v 1.0401 batch B and 11 fold of Bet v 1.0401 batch A to be necessary to trigger a similar mediator release when compared to Bet v 1.0101. So far, only conformation-dependent IgE epitopes have been identified on Bet v 1 [10]. Therefore, it seems likely that the documented conformational changes introduced in Bet v 1.0401 batch A by denaturation of the protein during recombinant production account for the abrogated IgE binding capacity of the molecule. Thus, we speculate that the observed IgE binding potential of batch B reflects the IgE binding capacity of natural Bet v 1.0401, whereas the low IgE binding activity of batch A seems rather artificial. Using allergen extracts for allergy diagnosis and therapy is associated with problems in extract standardization and often incontrollable batch-to-batch variability [27], [28]. The use of purified natural allergens to replace allergen extracts is also problematic, since natural allergen preparations can be contaminated with other allergenic molecules from the same source, and moreover, complex isoform combinations or post-translational modifications complicate the production of natural allergen preparations hindering the formulation of a standardized product. Therefore, the field of allergology is highly dependent on recombinant molecules and special attention should be paid on the quality of these products. The present study could demonstrate the influence of production procedures on the allergenic properties of an isoform of the major birch pollen allergen Bet v 1. The two different production methods had direct impact on primary and secondary structure of the allergen resulting in modified immunologic behavior. These results emphasize the necessity of certified biological reference preparations for allergenic products.
